# Time Course of Hemodynamic Responses to Different Doses of Lipopolysaccharide in Unanesthetized Male Rats

**DOI:** 10.3389/fphys.2019.00771

**Published:** 2019-06-25

**Authors:** Fernanda Brognara, Jaci Airton Castania, Daniel Penteado Martins Dias, Alexandre Kanashiro, Helio Cesar Salgado

**Affiliations:** ^1^ Department of Physiology, Ribeirão Preto Medical School, University of São Paulo, Ribeirão Preto, Brazil; ^2^ Barão de Mauá University Center, Ribeirão Preto, Brazil; ^3^ Department of Neurosciences and Behavior, Ribeirão Preto Medical School, University of São Paulo, Ribeirão Preto, Brazil

**Keywords:** lipopolysaccharide, arterial pressure, heart rate, heart rate variability, baroreflex sensitivity, unanesthetized rats

## Abstract

Lipopolysaccharide (LPS) administration is a well-known method to induce systemic inflammation widely used for investigating new therapeutic strategies for sepsis treatment, which is characterized by clinical manifestations such as tachycardia and hypotension. However, there are different doses of LPS used in several studies, and the hemodynamic responses were not always well characterized. Thus, the present study aimed to evaluate the arterial pressure, heart rate, heart rate variability, and baroreflex function from rats, over time, to different doses of LPS. Femoral artery and vein catheters were inserted into anesthetized Wistar-Hannover male rats for arterial pressure recording and LPS administration, respectively. On the next day, the arterial pressure was recorded before and after (90, 180, and 360 min) LPS injection (0.06, 20, 30, and 40 mg/kg). All doses of LPS tested increased the heart rate and decreased baroreflex sensitivity over time. In addition, while LPS administration of 20, 30, and 40 mg/kg increased the mean arterial pressure over time, 0.06 mg/kg decreased the mean arterial pressure at 360 min, as compared to baseline values. Furthermore, high doses of LPS decreased the power of the HF band of the cardiac interval spectrum over time, and the higher dose increased the power of the LF band. Our data indicate that high doses of LPS promote hypertensive response over time, while a low dose decreases arterial pressure. Moreover, the changes in heart rate variability and baroreflex function elicited by LPS may be not associated with arterial pressure response produced by the endotoxemia.

## Introduction

Models of endotoxemia have been used to explore the host innate immunity involved in the inflammatory response and searching therapeutic approaches for the treatment of inflammatory diseases (Borovikova et al., 2000; Bassi et al., 2015; Halbach et al., 2017). The well-known experimental model of endotoxemia elicited by lipopolysaccharide (LPS) administration has been widely used (Brognara et al., 2018; Chen et al., 2019). LPS is a component of the outer membrane of Gram-negative bacteria, which is a potent activator of the innate immunity, after being recognized by toll-like receptor 4 (Raetz and Whitfield, 2002; Beutler and Rietschel, 2003). This receptor is responsible for the activation of the nuclear factor-κB pathway that leads the synthesis and release of some pro-inflammatory mediators such as cytokines by macrophages (Beutler and Rietschel, 2003; Café-Mendes et al., 2017). Furthermore, this model of inflammation contributes for better understanding of the pathophysiological manifestations found in infection and inflammatory diseases, such as sepsis, mimicking clinical signs including hypo or hyperthermia, tachycardia, hypotension, and tachypnea (Cai et al., 2010; Café-Mendes et al., 2017; Komegae et al., 2018; Wasilczuk et al., 2019).

The literature indicates that different doses of LPS have been used in several studies, but the hemodynamic responses involved are not always shown or even considered. Usually, LPS administration promotes hypotension and tachycardia; but, in the literature, these responses are not precisely described concerning the different doses employed, especially in unanesthetized animals. Therefore, the present study aimed to evaluate the arterial pressure, heart rate, and the heart rate variability responses from unanesthetized rats, over time, to different doses of LPS.

## Materials and Methods

### Experimental Animals

The experiments were performed on male Wistar-Hannover rats weighing 210–280 g obtained from the Main Animal Facility of the University of São Paulo (Campus of Ribeirão Preto; Ribeirão Preto, SP, Brazil), maintained under controlled temperature (22°C) and constant 12 h light-dark cycle, and provided with food and water ad libitum. All procedures were reviewed and approved by the Committee of Ethics in Animal Research of the Ribeirão Preto Medical School – University of São Paulo (protocol number 194/2016).

### Surgical Procedures

Animals were anesthetized with a cocktail of ketamine and xylazine (50 mg/kg and 10 mg/kg, i.p.) and then subjected to surgical procedures to catheterization of the femoral artery and vein for arterial pressure recording and LPS administration, respectively. Briefly, the left femoral artery was catheterized with polyethylene tubing (PE-50 soldered to PE-10 polyethylene tube; Intramedic, Clay Adams, Parsippany, NJ, USA) for arterial pressure recording. The catheter implanted into the femoral artery was filled with 100 IU/ml heparin in saline. Additionally, a catheter (PE-50 polyethylene tube; Intramedic, Clay Adams, Parsippany, NJ, USA) was inserted into the left femoral vein for the administration of LPS from *Escherichia coli* 0111: B4 purified by phenol extraction (Sigma-Aldrich, St. Louis, MO, USA). The catheters were pulled up through a subcutaneous track to the animal’s neck and exteriorized in the back of the nape, and the surgical incisions were sutured. Analgesic (tramadol hydrochloride: 2 mg/kg, s.c.) was injected immediately after the end of surgery.

### Arterial Pressure Recording

To record the pulsatile arterial pressure, the arterial catheter was connected to a pressure transducer (MLT844; ADInstruments, Bella Vista, Australia) and the signal was amplified (ML224; ADInstruments, Bella Vista, Australia) and sampled at 2 kHz by an IBM/PC computer (Core 2 Duo, 2.2 GHz, 4 GB RAM) attached to an analog-to-digital interface (PowerLab, ADInstruments, Bella Vista, Australia). The experiment was conducted with the animals moving freely in their own cage (one rat per cage) and silence was maintained to minimize environment stress. Arterial pressure recordings were processed with computer software (LabChart 7.0, ADInstruments, Bella Vista, Australia) capable of detecting inflection points and generate mean arterial pressure and heart rate beat-by-beat time series.

### Experimental Procedures

Twenty-four hours after the surgical procedures, the rats were assigned into five groups with different doses of LPS: (1) 0.06 mg/kg (*n* = 7), (2) 20 mg/kg (*n* = 4), (3) 30 mg/kg (*n* = 3), (4) 40 mg/kg (*n* = 5), and (5) with saline administration (control, *n* = 5). With the unanesthetized animals moving freely, the experimental protocol consisted of basal recordings of pulsatile arterial pressure, followed by administration of LPS or saline. The arterial pressure was also recorded 90, 180, and 360 min after LPS or saline injection to evaluate the time course of the hemodynamic parameters.

### Heart Rate and Systolic Arterial Pressure Variability Analysis

Beat-by-beat time series with systolic arterial pressure and cardiac interval values were extracted from periods of approximately 10 min from each moment (basal, 90, 180, and 360 min after LPS or saline) from pulsatile arterial pressure tracings. The series were analyzed in the frequency domain by means of spectral analysis using open access custom computer software (CardioSeries v2.7, www.danielpenteado.com). Briefly, the beat-by-beat time series were resampled using cubic spline interpolation (10 Hz) and the interpolated series were split in half-overlapping sequential segments of 512 data points. All segments were visually inspected looking for transients that could affect the calculation of the power spectral density (Fast Fourier Transform), and the spectra were integrated in low- (LF: 0.20–0.75 Hz) and high-frequency (HF: 0.75–3.0 Hz) bands. Results are expressed in absolute (ms^2^) and normalized (nu) units. LF/HF ratio was also calculated.

### Baroreflex Sensitivity Analysis

The same beat-by-beat time series selected for heart rate and systolic arterial pressure variability analysis were used for assessment of spontaneous baroreflex sensitivity. In the time domain, analysis was carried out using the sequence method (Bertinieri et al., 1985; Di Rienzo et al., 1985). Time series were scanned by the CardioSeries software, searching for sequences of data values with at least four consecutive beats in which rises in systolic arterial pressure were followed by cardiac interval lengthening and decreases in systolic arterial pressure were followed by cardiac interval shortening, with a calculated linear correlation higher than 0.85. The average of the slopes of the linear regression lines between systolic arterial pressure and cardiac interval values was taken as the baroreflex sensitivity. In addition, spontaneous baroreflex sensitivity was assessed into the frequency domain using cross-spectral analysis through the Fast Fourier Transform. In brief, beat-by-beat time series were interpolated (10 Hz), split into half-overlapping sequential sets of 512 points, and had cross-spectra calculated. When a coherence function returned values greater than 0.5, the average gain of the transfer function into the LF band (0.20–0.75 Hz) was taken as a measure of the baroreflex gain.

### Statistical Analysis

The data were analyzed by one-way analysis of variance and one-way analysis of variance for repeated measures followed by a Student-Newman-Keuls’s or Dunn’s *post hoc* test when indicated. Differences were considered statistically significant if *p* < 0.05. The results are shown as the mean ± standard error of the mean. Statistical analysis was performed using SigmaPlot 12.0 software (Systat Software, San Jose, CA, USA).

## Results

### Time Course of the Arterial Pressure to Lipopolysaccharide Administration

**Figure 1:**
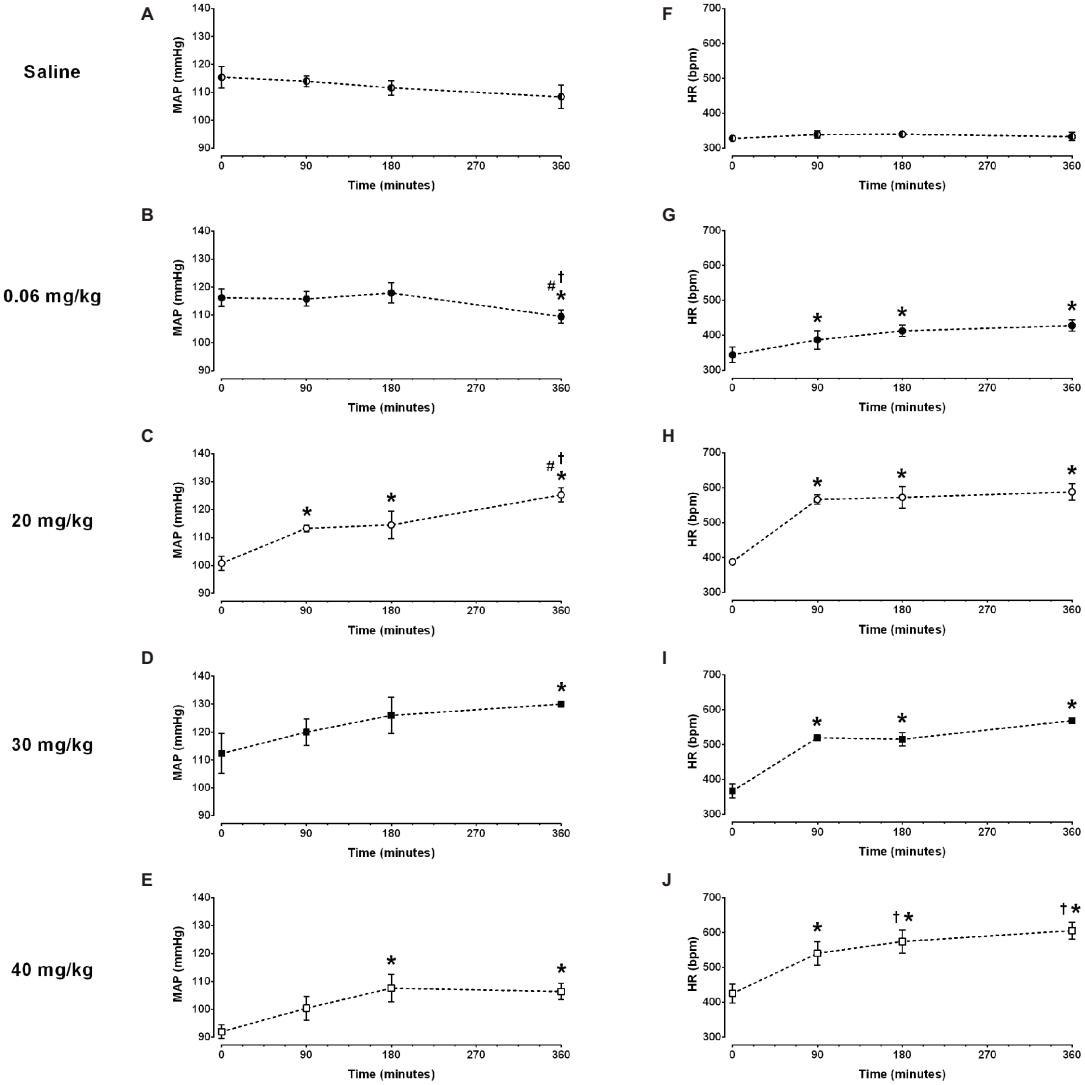
Time course of mean arterial pressure (MAP) and heart rate (HR) response to different doses of lipopolysaccharide in unanesthetized rats. Saline: panels **(A,F)**; 0.06 mg/kg of LPS: panels **(B,G)**; 20 mg/kg of LPS: panels **(C,H)**; 30 mg/kg of LPS: panels **(D,I)**; 40 mg/kg of LPS: panels **(E,J)**. Data are expressed as mean ± standard error of the mean. ^*^p < 0.05 vs. baseline counterpart; ^†^*p* < 0.05 vs. 90 min counterpart; ^#^*p* < 0.05 vs. 180 min counterpart.

No changes in mean arterial pressure were observed over time in the saline group (Figure 1A and Table 1). As compared to baseline, the dose of 0.06 mg/kg of LPS decreased the mean arterial pressure at 360 min after its administration (116 ± 3 vs. 109 ± 2 mmHg, *p* = 0.025; Figure 1B and Table 1). In contrast, the doses of 20, 30 and 40 mg/kg increased the mean arterial pressure over time (Figures 1C–E and Table 1). The doses of 20 and 30 mg/kg reached the hypertensive peak at 360 min and the dose of 40 mg/kg at 180 min (Figures 1C–E and Table 1). In addition, the dose of 20 mg/kg was able to increase the mean arterial pressure already in the first 90 min after LPS, while the doses of 40 and 30 mg/kg were only able to increase the arterial pressure 180 and 360 min, respectively (Figures 1C–E and Table 1).

**Table 1:** Time course of mean arterial pressure (MAP) and heart rate (HR) response to different doses of lipopolysaccharide.

	Saline (*n* = 5)	0.06 mg/kg (*n* = 7)	20 mg/kg (*n* = 4)	30 mg/kg (*n* = 3)	40 mg/kg (*n* = 5)
**MAP (mmHg)**										
Baseline	115±4	116±3	101±2	112±7	92±2
90 min	114±2	116±3	113±1^*^	120±5	100±4
180 min	112±3	118±4	114±5^*^	126±6	108±5^*^
360 min	108±4	109±2^*^ ^,^ ^†^ ^,^ ^#^	121±4^*^ ^,^ ^†^ ^,^ ^#^	130±0.4^*^	107±3^*^
**HR (bpm)**										
Baseline	328±5	344±22	388±6	367±21	426±27
90 min	339±10	387±27^*^	566±13^*^	520±6^*^	540±34^*^
180 min	340±8	413±16^*^	572±31^*^	516±18^*^	574±33^*^ ^,^ ^†^
360 min	333±12	428±17^*^	588±24^*^	569±9^*^	606±24^*^ ^,^ ^†^

Data are expressed as mean ± standard error of the mean.

* *p* < 0.05 vs. baseline counterpart;

† *p* < 0.05 vs. 90 min counterpart;

# *p* < 0.05 vs. 180 min counterpart.

### Time Course of the Heart Rate to Lipopolysaccharide Administration

No changes in heart rate were observed over time in the saline group (Figure 1F and Table 1). All doses of LPS tested in this study increased the heart rate over time, compared to baseline values and reached a peak at 360 min after LPS administration Figures 1G–J and Table 1). Nevertheless, the dose of 0.06 mg/kg promoted a smaller tachycardia as compared to the other doses evaluated (0.06 mg/kg: Δ 84 ± 23 bpm vs. 20 mg/kg: Δ 200 ± 26 bpm, *p* = 0.007; 30 mg/kg: Δ 202 ± 29 bpm, *p* = 0.023; 40 mg/kg: Δ 180 ± 29 bpm, p = 0.007). Moreover, all doses of LPS tested were able to increase the heart rate already in the first 90 min after their administration (Figures 1G–J and Table 1).

### Time Course of the Heart Rate Variability to Lipopolysaccharide Administration

**Figure 2:**
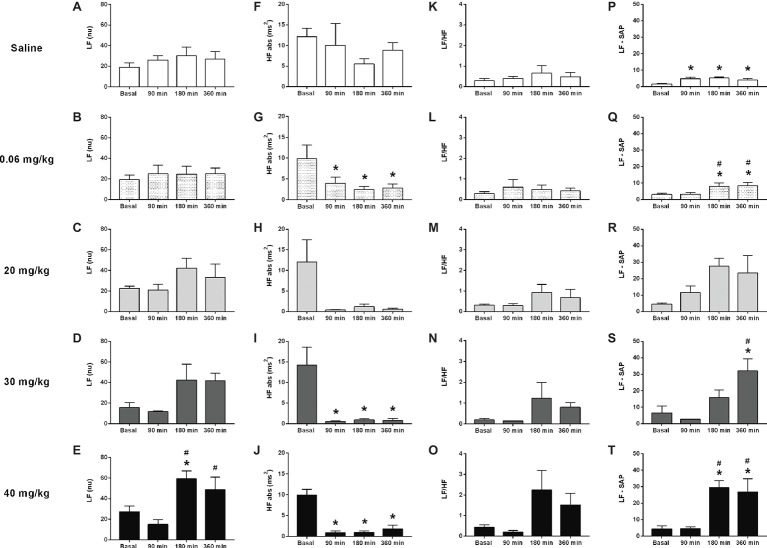
Effects of different doses of lipopolysaccharide in the heart rate variability from unanesthetized rats. The heart rate variability parameters are the following: LF band (LF), HF band (HF), LF/HF ratio, and LF from the systolic arterial pressure (LF-SAP). Saline: panels **(A,F,K,P)**; 0.06 mg/kg of LPS: panels **(B,G,L,Q)**; 20 mg/kg of LPS: panels **(C,H,M,R)**; 30 mg/kg of LPS: panels **(D,I,N,S)**; 40 mg/kg of LPS: panels **(E,J,O,T)**. Data are expressed as mean ± standard error of the mean. ^*^p < 0.05 vs. baseline counterpart; ^#^*p* < 0.05 vs. 90 min counterpart. LF, low frequency; HF, high frequency.

The analysis performed over several time points (basal, 90, 180, and 360 min) following LPS administration, revealed no differences in the power of the LF band of the cardiac interval spectrum in saline, 0.06, 20, or 30 mg/kg LPS groups, despite a strong trend toward enhanced LF power in 20 or 30 mg/kg groups (Figures 2A–D). However, the rats that received 40 mg/kg showed an increased LF power of the cardiac interval spectrum at 180 and 360 min following LPS administration (Figure 2E). The analysis of HF power depicted no changes in the saline group (Figure 2F); but LPS injection drastically reduced the HF power over time, starting at 90 min after its administration (Figures 2G–J). In all groups, no differences were found in the LF/HF ratio over time (Figures 2K–O). Nevertheless, the dose of 40 mg/kg presented a great trend toward an increase at 180 and 360 min after LPS (Figure 2O). The power of the LF band of systolic arterial pressure spectra increased over time in either, saline and LPS treated groups (Figures 2P–T).

### Time Course of the Baroreflex Sensitivity to Lipopolysaccharide Administration

**Figure 3:**
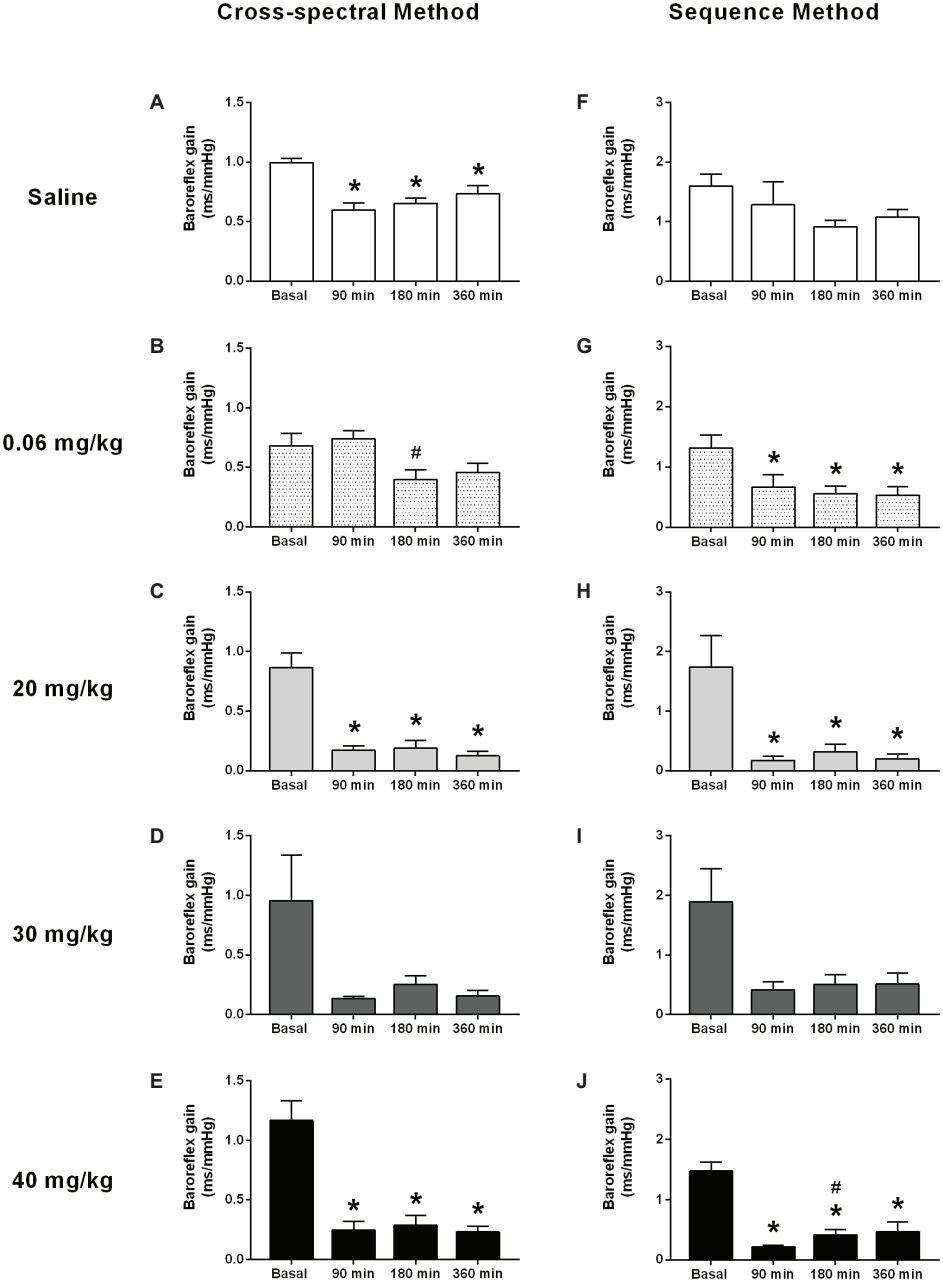
Effects of different doses of lipopolysaccharide in the baroreflex sensitivity in unanesthetized rats. Baroreflex sensitivity analysis by cross-spectral (panels **(A–E)**) and the sequence method (panels **(F–J)**), after saline or LPS administration. Saline: panels **(A,F)**; 0.06 mg/kg of LPS: panels **(B,G)**; 20 mg/kg of LPS: panels **(C,H)**; 30 mg/kg of LPS: panels **(D,I)**; 40 mg/kg of LPS: panels **(E,J)**. Data are expressed as mean ± standard error of the mean. ^*^p < 0.05 vs. baseline counterpart; ^#^*p* < 0.05 vs. 90 min counterpart.

In general, both methods of analysis (i.e., cross-spectral analysis and the sequence method) revealed a decrease in baroreflex sensitivity in all LPS groups over time (Figures 3B–E,G–J). This change was observed early in the first moment evaluated (90 min) and was kept until the end of the protocol (360 min). In the saline group, no changes were observed over time, when baroreflex sensitivity was evaluated by the sequence method (Figure 3F); while a slight reduction was shown by the cross-spectral analysis approach (Figure 3A).

## Discussion

In the current study, we described the changes in arterial pressure and heart rate as well as the heart rate variability, over time, elicited by different doses of LPS administration. Concerning the heart rate, we observed that all doses tested increased this hemodynamic parameter, over time, supporting previous observation (Altavilla et al., 2002). Moreover, in agreement with previous studies (Martelli et al., 2019), our findings demonstrated that the lowest dose tested (0.06 mg/kg) reduces the mean arterial pressure over time. However, it has been described a transient increase in mean arterial pressure, 2 and 3 h after LPS administration, with the return of this hemodynamic parameter to baseline, 4–6 h later, using the same dose (0.06 mg/kg) (Martelli et al., 2014). Apropos, this dose of LPS has been extensively used in rats for investigating the mechanisms involved in the inflammatory reflex pathway (Martelli et al., 2014, 2019; Komegae et al., 2018), as well as for evaluating the baroreflex regulation during systemic inflammation (Tohyama et al., 2018).

Previous studies using higher doses of LPS, for eliciting the inflammatory process, demonstrated that this approach promoted a hypotensive response (Altavilla et al., 2002; Doursout et al., 2016). For instance, Dourtsout and coworkers (Doursout et al., 2016) observed a decrease in mean arterial pressure, 1 h after the administration of a high dose of LPS, in conscious rats, followed by the recover to baseline levels 2 and 3 h later. Another study evaluated the time course of the mean arterial pressure for 360 min, after LPS administration, displaying a sustained reduction of this hemodynamic parameter in anesthetized rats, over time, initiating 30 min after the injection (Altavilla et al., 2002). However, the current study did not provide support to this finding, since the intravenous administration of higher doses of LPS (20, 30, and 40 mg/kg) did not reduce the mean arterial pressure in unanesthetized rats, but increased over time, starting sometimes 90 min after their administration (as was the case of the dose of 20 mg/kg). Many factors can influence the inflammation-induced cardiovascular alterations that could explain these discrepancies between the studies, deserving an investigation in the future, including the gender of the subjects, quality of the LPS (i.e., purity, source, and route of administration) and rat strains (Sternberg et al., 1989; Simons et al., 1998; Marriott et al., 2006; Ferguson et al., 2013).

It is noteworthy to mention that some studies report changes in the arterial pressure after administration of intermediate (i.e., 5 and 10 mg/kg) doses of LPS in unanesthetized rats. Mehanna and coworkers (Mehanna et al., 2007) described three phases for the arterial pressure response within the first hour after LPS (5 mg/kg) which consisted of an initial decrease (phase 1), rebound recovery (phase 2), and a long-lasting decrease (phase 3). Other studies also showed that the dose of 10 mg/kg promoted a reduction in arterial pressure over time (i.e., up to 180 min) (Sallam et al., 2017, 2018). Nevertheless, Lee et al. (2005) in an extended analysis (i.e., up to 24 h after LPS administration), observed that the arterial pressure changes consisted of an initial hypotensive response (i.e., at 30–60 min), followed by a hypertensive response (i.e., from 1 to 9 h), and finally by a second hypotensive response (i.e., from 9 to 24 h) after the administration of 5 mg/kg of LPS in unanesthetized rats. Thus, it seems that the response of the arterial pressure to LPS is quite variable over time, and several elements may influence this outcome, as mentioned previously.

In addition, the results of the present study clearly showed that higher doses of LPS increased the power of the LF band and decreased the power of the HF band spectra over time (i.e., following LPS administration). In other words, LPS enhanced the sympathetic tone and reduced the vagal tone in unanesthetized rats. It is well known that LPS administration influences the heart rate variability of rats (Huang et al., 2010; Zila et al., 2015). Nevertheless, to the best of our knowledge, no previous study has described the effects of different doses of LPS on heart rate variability, over time, in unanesthetized rats. Since all LPS-treated groups (i.e., low and higher doses) showed a decrease in the HF power (i.e., reduction of the vagal tone), and only the lower dose diminished the arterial pressure over time, while the others promoted the opposite response, it seems that the hypotensive response elicited by the lower dose of LPS is not dependent of an increase, or maintenance, of the vagal tone over time. However, differences observed in the arterial pressure response among groups injected with high and low doses of LPS could be attributed to the exacerbated increase in the power of the LF band of the systolic arterial pressure over time in the groups received high doses of LPS, since this parameter is related to peripheral resistance of blood vessels (Julien, 2006). Thus, the enhanced peripheral resistance may have contributed to the hypertensive response from the high LPS dose groups.

The baroreflex function has been explored after LPS administration (Shen et al., 2004; Radaelli et al., 2013; Tohyama et al., 2018). In the present study, the analysis of the baroreflex function revealed a decrease in baroreflex sensitivity in all LPS-treated groups, over time, regardless of the dose used. This impairment was observed early in the first moment and was sustained until the end of the protocol. In agreement with our results, Radaelli et al. (2013) showed a sustained reduction in baroreflex sensitivity, which starting right after the beginning of the infusion of LPS in rats (i.e., 10 min later). Furthermore, Shen et al., (2004) concluded that an efficient arterial baroreflex is essential to determine survival during the LPS-induced lethal shock. Baroreflex impairment has meaningful consequences in arterial pressure because, under the absence of the reflex control of arterial pressure, the cardiovascular system loses the ability to control appropriately the arterial pressure, leading to hypotensive or hypertensive responses during endotoxemia. Since all groups receiving LPS showed impairment of baroreflex function over time, correlations between baroreflex impairment and arterial pressure changes cannot be made, since rats receiving a low dose of LPS had a decrease in arterial pressure, while the rats receiving high doses of LPS had an increase in arterial pressure.

In conclusion, our data show that while opposite changes of the arterial pressure responses are dependent on the dose of LPS administrated, the tachycardic response seems to be independent of the dose. We suggest that changes in the heart rate variability and baroreflex function elicited by LPS may not be associated with the arterial pressure response produced by the endotoxemia.

## Data Availability

All datasets generated for this study are included in the manuscript and/or the supplementary files.

## Ethics Statement

This study was carried out in accordance with the recommendations of Committee of Ethics in Animal Research of the Ribeirão Preto Medical School – University of São Paulo. The protocol was approved by the Committee of Ethics in Animal Research of the Ribeirão Preto Medical School – University of São Paulo (protocol number 194/2016).

## Author Contributions

FB conceived and designed the research study, performed experiments, analyzed data, interpreted results, prepared figures and table, and wrote the manuscript. JC performed experiments. DD analyzed data, commented, and edited the manuscript. AK and HS commented and edited the manuscript. All the authors reviewed and approved the final version of manuscript before submission.

### Conflict of Interest Statement

The authors declare that the research was conducted in the absence of any commercial or financial relationships that could be construed as a potential conflict of interest.
